# The Effect of Positive Emotion and Interpersonal Relationships to Adaptation of School Life on High School Athletic Class Students

**DOI:** 10.3390/ijerph17176354

**Published:** 2020-08-31

**Authors:** Chia-Fu Chang, Huey-Hong Hsieh, Hsiu-Chin Huang, Yu-Lan Huang

**Affiliations:** 1 Physical Education and Arts School, Chengyi University College, Jimei University, Xiamen 361023, China; aa760392@yahoo.com.tw; 2 Department of Leisure Management, Taiwan Shoufu University, Tainan 72153, Taiwan; nancylin809@gmail.com; 3 Department of Physical Education, Health & Recreation, National Chiayi University, Chiayi 62103, Taiwan; eesb7606@yahoo.com.tw

**Keywords:** high school athletic class, positive emotion, interpersonal relationships, adaptation of school life

## Abstract

Background: Adaption for school life is important for all students. As for athletic students, since they need to cope with schoolwork and extensive training, adaption for school life could be very challenging. Taking this into consideration, the purpose of this study was to explore the factors which may help high school athletic students’ adaption of school life. Owing to this, the study explored previous researches and proposed four hypotheses: the first two hypotheses proposed that athletes’ positive emotion will have positive impacts on both their interpersonal relationships and adaption of school life; the third hypothesis suggests that athletes’ interpersonal relationships will have positive impacts on their adaption of school life and the fourth hypothesis suggested that interpersonal relationships play a mediating role among the positive emotion’s effect on adaption of school life. Methods: A total of 800 structured questionnaires were distributed to eleven high schools with athletic class students for data collection with a valid return rate of 90.6%. Structural equation modelling was used to test the relationship among them. Results: The result showed that positive emotion (β = 0.72, *p* < 0.05) and interpersonal relationships (β = 0.34, *p* < 0.05) had positive impacts on students’ adaption of school life with a predictive power of 68%. In addition, positive emotion also affected students’ school life adaption through interpersonal relationships. Conclusion: The study confirmed the positive emotion can have significant influences on student athletes’ interpersonal relationships and school life adaption. Implications: According to our findings, we suggest to encourage and promote athletes’ positive emotions so to help them have better interpersonal relationships and school life adaption.

## 1. Introduction

Athletic classes are established to discover and develop talented student athletes, and to deliver a training environment where athletes learn the best skills to compete in any organized competition [1,2]. However, full-time high school athletic class students usually have a hard time balancing academic achievement and sports engagement, and thus are more likely to be exposed to stress. According to Chen, Lin and Lin [3], stress that is not handled properly can negatively affect student athletes’ psychological well-being and hinders their athletic performance, or even worse, result in students giving up their academic learning. Especially to students who dedicate much of their time to sports, they may get trapped in a vicious circle of struggling balancing school with sports and studying, chances are they can have more problems with their future career options. As a result, it is very important for high schools to develop a mechanism to help students handle stress and improve their mental resilience. In recent years sports research has explored impacts of positive psychology on athlete performance [4]. Positive psychology has been used to handle a complex array of negative emotions, such as stress, depression, anxiety, and distress [5].

Research on positive psychology asserted that a positive support system helps trauma recovery. It also has healing power for people facing stress in their daily lives [6]. Studies showed the increase of positive energy minimizes the impacts of negative mentality [6]. Bandura [7], Deci and Ryan [8], Hanin [9] used emotions and motivations as predictors to study those effects on athletic performance. Their study found emotions and motivations were highly correlated [8] and athletes with higher involvement motivations generated positive emotions. In Hanin’s individualized zones of optimal functioning model [9], he pointed out that personnel with proper emotion status would have the best performance in sports. From previous studies, we can see that people with positive mentality bring up good achievements. Therefore, this present study explored the effects of positive mentality of high school athletic class students on the adaption to their school life.

As for high school student athletes’ interpersonal relationships, since high school athletic class students usually have a separate curriculum because they have training programs to follow, they do not necessarily follow the regular school schedule. This circumstance will allow them to have more personal time to be able to use it in activities that contribute to their socialization; however, their social connections can be limited in a small group [10] and their interpersonal relationships may also affect their adaption of school life. Therefore, this study would also explore the effects of interpersonal relationships on high school student athletes’ adaption of school life.

In the sports field, especially in oriental society, being an athlete is not considered as being a “winner”, and unless you get prizes in international games, in general, it is not easy to get a good job in the future. Therefore, parents still want their children to have school success. It adds burden to athletes. Few studies discuss high school athletic students’ coping with both training stress and school stress. Therefore, we hope this study can contribute to society to assist high school athletic students to cope with those situations and continue to love sports and retain the zeal as athletes.

## 2. Literature Review and Conceptual Framework Development

### 2.1. The Relationship between Positive Emotions and Interpersonal Relationships

Fredrickson [11] proposed the broaden-and-build theory, which suggests that positive emotion broadens individual’s awareness, encourages exploratory thoughts and actions, and builds individual’s personal resources, such as physical and mental health, social connection, and positive behaviors and attitudes. All these resources built up over time increase the individual’s ability to cope with challenging situations and adversity. In fact, interpersonal relationships, social communication, demographic background, and personal traits all play a part in building up personal resources. By contrast, emotional swings, weak social skills and communication restrict individual’s social networking and disconnect their sense of belonging [12,13].

Studies have shown that emotion affects behavior. People having positive emotions are welcomed in any given group [12,13]. A study by Chang, Lin, and Yeh [14] demonstrated that positive emotions, such as joy, satisfaction, happiness, bring out genuine, active and positive behaviors. People with positive emotions are contagious, are likely to share enjoyment, and are thus mostly welcomed. Similarly, Huang [15] revealed that students who participate in after-class sports clubs are more likely to be mentally positive, have healthier peer relationships, and have more career options, as compared with those who do not participate in after-class activities. It is also reported that positive emotions lead to successful interpersonal relationships. As a result, the research hypothesis 1 was formulated as follows:

**Hypothesis** **1** **(H1).** *High school athletic students’ positive emotions have a positive impact on their interpersonal relationships.*

### 2.2. The Relationship between Positive Emotions and Adaptation of School Life

Friend support and conflicts with friends in adolescence have some effects on developmental tasks, such as personal tasks like acquisition of autonomy in organization of leisure time, social-institutional tasks, in term of school success [16]. In other words, participation in social and interpersonal relationships helps to provide the resources for successfully coping with developmental tasks in adolescence [17]. Therefore, adaption of school life is important for the success of adolescences in coping with developmental tasks.

Positive emotion is a subjective well-being that makes people open to new ideas and actions and more adaptive to the surrounding environment [11]. Positive emotions are typically contagious. Happy people interact with others better, and face challenges with a positive attitude, and they can expect to be treated the same way. They create a positive cycle, which helps them successfully adapt to new life. Hong [18] declared that an individual’s positivity, due to different life experiences, changes over time. Research has suggested that positive mentality is positively related to adaptation to life, and the fact that students typically associate positive emotions with good influence on their lives resonated with the research findings. As for athletes, Lin [19] discovered that athletes who better develop psychological skills are more capable of adapting to new life, because psychological skills enhance their well-being in sports. Lo, Guu, and Tseng [13] declared that experimental group students who joined six-week positive emotion course scored higher on life-adaptability post-test than control group students who participated in a course with integrated common activities. Similar results were found for the follow up test three weeks after the conclusion of the courses, and suggested that positive emotion has continuous effects on students’ adaptability in school. Drawing from previous findings, positive emotions were found to have positive effects on adolescents’ adaptability to life. Therefore, this study proposes the following hypothesis:

**Hypothesis** **2** **(H2).** *High school athletic students’ positive emotions have positive impacts on their adaptation of school life.*

### 2.3. The Relationship between Interpersonal Relationships and Adaptation of School Life

Strong interpersonal relationships are an indication that an individual has competence facilitating interaction and communication with others. It is essential for everyone in a society, and it may indirectly affect an individual’s social status [20]. In adolescence peer relationship appears to the most important relationship for students as they spend most time in school. A good peer relationship indicates that students are more easily able to adapt to the school setting [21].

In addition, Wu [22] examined the relationship among positive emotions, interpersonal problem-solving attitudes, and school life adjustment of junior high school students. Results of his study revealed a significant positive effect between positive emotions and school life adjustment, and between interpersonal problem-solving attitudes and school life adjustment. Results also showed that interpersonal problem-solving attitudes are a strong predictor of school life adjustment. Since peer relationship is an indicator of how well students interact with each other and how well they adapt to school environment, it contributes to a culture which encourages sharing and interaction, which in turn, makes students feel comfortable in school [20]. Based on previous studies, we proposed the following:

**Hypothesis** **3** **(H3).** *High school athletic students’ interpersonal relationships have a positive impact on their adaptation of school life.*

### 2.4. The Mediating Effect of Interpersonal Relationships among Positive Emotions and Adaptation of School Life and Adaptation of School Life

Based on the proposed H1, H3, we can see that both positive emotions and interpersonal relationships have positive effects on adaption of school life and from H2, we can see positive emotions also have direct effects on interpersonal relationships, therefore, we can infer that interpersonal relationships play the mediation role among the effects of positive emotions to adaption of school life from the postulated hypotheses H1, H2 and H3. Therefore, hypothesis H4 is proposed as the following:

**Hypothesis** **4** **(H4).** *High school athletic students’ positivity will affect their adaption of school life through interpersonal relationships.*

**Figure 1:**
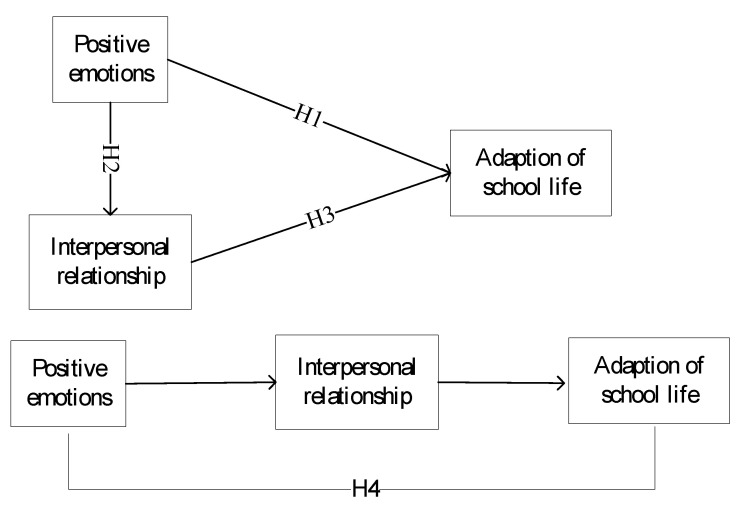
Research hypotheses.

Figure 1 present the research hypotheses.

## 3. Methods

### 3.1. Data Collection

Research subjects were freshmen, sophomore, and senior student athletes from senior high schools across Taiwan. Eleven schools were selected based on the relative proportions of the number of schools distributed in four geographic areas, respectively (four from the north, three from the central, three from the south, and one from the east). Following the school selection, the researcher made phone calls and explained the purpose of the study. With the school approval, the researcher sent survey questionnaires to each school and asked the school directors to give questionnaires to class instructors and sports coaches, who later asked students to fill out the questionnaires and gatherer them back. A total of 800 questionnaires were distributed and 725 questionnaires were collected for data analysis, with a valid return rate of 90.6%.

### 3.2. Measurements

Background variables were gender, grade in school, year of sports experience, and sports expertise.

The positive emotion scale was adopted from Lee, Chien, and Lee [22], and included three dimensions: (1) joy, (2) satisfaction, and (3) flow. The scale consisted of 12 items and were measured by a 5-point Likert scale, ranging from 1 = strongly disagree to 5 = strongly agree.

The interpersonal relationships scale was a modified version of Huang [23], which was consisted of two aspects including (1) social skills and (2) peer attachment. Subjects rated each of the seven items using a 5-point scale in Likert format, 1 = strongly disagree to 5 = strongly agree.

The adaptation of school life scale was modified from Huang [23] and Wu [24]. The scale was comprised of three aspects: (1) peer relationship, (2) learning adjustment, and (3) teacher-student relationship. Fifteen items were measured by a 5-point Likert scale, ranging from 1 = strongly disagree to 5 = strongly agree.

This study adopted a partial least squares model and the Warp PLS version 5.0 statistical software (WarpPLS, Laredo, TX, USA) developed by Kock [25] to verify all scales’ validity and reliability. According to the suggestion by Hulland [26], an analysis of the validity and reliability of all relevant scales in a model shall examine reliability, convergent validity, and discriminant validity.

In Table 1, According to Fornell and Larcker [27], the composite reliability and the Cronbach’s α were acceptable if they were equal to or greater than 0.70. In this present study, the composite reliability and the Cronbach’s α all exceeded 0.70, showing the reliability of each scale was acceptable.

Convergent validity, the factor loading of assessed items on joy dimension of the positive emotion scale exceeded 0.50 and fell between 0.734 and 0.889. The factor loading of items of satisfaction dimension fell within 0.797 and 0.846 (>0.50). The factor loading of items of the flow dimension was between 0.746 and 0.836 (>0.50). Regarding to the interpersonal relationships scale, the factor loading of assessed items of social skills dimension was between 0.786 and 0.805 (>0.50), and that of peer attachment dimension fell within 0.736 and 0.893 (>0.50). On adaptation of school life scale, the factor loading of peer relationship aspect was between 0.782 and 0.874 (>0.50), and that of the learning of adjustment aspect fell within 0.699 and 0.839 (>0.50). The factor loading of teacher-student relationship dimension fell between 0.851 and 0.890 (>0.50). That factor loadings of the study variables were all greater than the acceptable standard [28], indicating a good convergent validity.

Discriminant validity, according to Chin [29], is assessed by demonstrating the average variances extracted (AVE) among the latent variables. To assess discriminant validity in this manner, the AVE should be equal to or greater than 0.50. This is determined by comparing the square root of the AVE to the correlation of the latent variables. Additionally, Venkatesh, Thong, and Xu [30] suggested that the square root of the AVE of all latent variables must be equal to or greater than 0.50.

As shown in Table 1, the square root of the AVE of all latent variables exceeded 0.50 and fell between 0.797 and 0.867, and was also higher than correlation coefficients in the same column and row of the same construct. It is evident that the measurement model has demonstrated a very good discriminant validity.

## 4. Results

### 4.1. Demographic Analysis Results

Table 2 presents the demographic statistics of the participants. Research participants were 600 (82.8%) males and 125 (17.25) females, most of whom was freshman (N = 289, 39.6%), followed by sophomore (N = 226, 31.2%) and senior (N = 212, 31.2%). The majority (N = 420, 57.9%) had six years of sport experience, followed by those with 4–5 years of experience (N = 167, 23%), with 2–3 years (N = 100, 13.8%) and with 1 year (N = 38, 5.2%). A total of 446 (61.5%) were ball game participants, 27 (3.7%) were choreography dancers, 46 (6.3%) participated in equipment requiring sport, and 206 (28.4%) participated in competitive sports.

### 4.2. Hypotheses Tests

**Figure 2:**
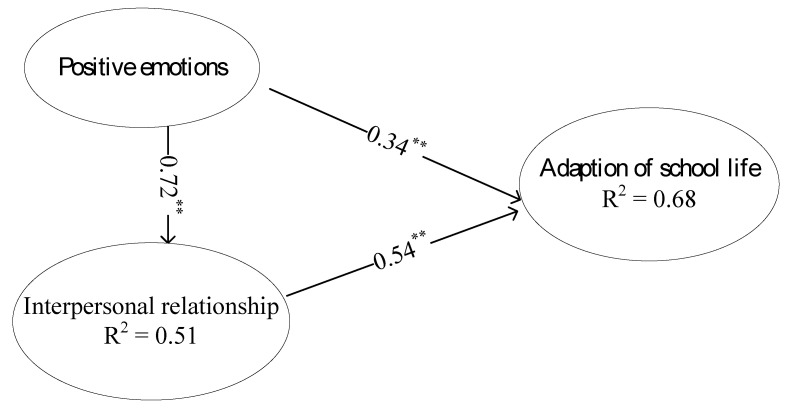
Standardized parameter estimation of overall model. Note: ** *p* < 0.01.

**Figure 3:**
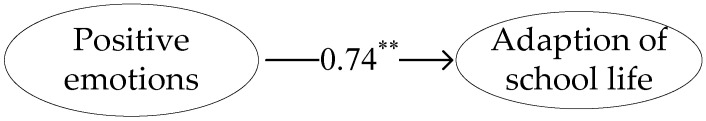
PLS results of the relationship between positive emotion and adaptation of school life. Note: ** *p* < 0.01.

All hypotheses were tested and the path coefficients were presented in Figure 2 and Figure 3.

H1: High school athletic students’ positive emotions have a positive impact on interpersonal relationships. Results from PLS showed that the effect of positive emotions on interpersonal relationships among high school athletic class students was significant (β_1_ = 0.72, *p* < 0.05), which indicated that the higher positive emotions led to better interpersonal relationships.

H2: High school athletic students’ positive emotions have positive impacts on the adaptation of school life. Results from PLS showed that effect of positive emotions on school adaptation among high school athletic class students was significant (β_2_ = 0.34, *p* < 0.05), which indicated that the higher level of positive emotions resulted in better school adaptation.

H3: High school athletic students’ interpersonal relationships have a positive impact on the adaptation of school life. Results from PLS showed that the effect of interpersonal relationships on adaptation of school life was significant (β_3_ = 0.54, *p* < 0.05), which indicated that better interpersonal relationships resulted in better school adaptation.

H4: High school athletic students’ positive emotions will affect their adaption of school life through interpersonal relationships. Results from PLS showed that the mediating effect of interpersonal relationships between positive emotions and school adaptation was found to be significant (β_4_ = 0.39, *p* < 0.05), suggesting that positive emotions had indirect effect on school adaptation through interpersonal relationships. Figure 2 and Figure 3 showed that path coefficient of positive emotions on school adaptation decreased from 0.74 (*p* < 0.05) to 0.34 (*p* < 0.05). According to Baron and Kenny [31], complete mediation is present when the independent variable no longer influences the dependent variable after the mediator has been controlled, indicating statistical significance is not found to be exist. Partial mediation occurs when the independent variable’s influence on the dependent variable is reduced, and statistical significance is however reported, after the mediator is controlled. In this current study, interpersonal relationships were a partial mediator.

### 4.3. Explanatory Power

R^2^ value means prediction power of research model. It is the percentage of the variance of the endogenous explained by all exogenous. High value indicates a better predictability. In Figure 2, positive emotion explained 51% of the overall variance of interpersonal relationships of high school athletic class students. Figure 2 also showed that positive emotion and interpersonal relationships together explained 68% of all variance of school adaptation.

## 5. Discussion

### 5.1. The Relationship between Positive Emotions and Interpersonal Relationships

According to the hypothesis test, high school athletic students’ positive emotions have a positive impact on interpersonal relationships. Seligman [32] stated that positive emotions bring enjoyment to the daily routine and act as buffers against setbacks. Positive emotions are contagious, and give the big boost of the well-being of others [33]. Overall, positive emotions provide powerful benefits to individuals [9].

Emotional contagion theory explains that emotions can be transferred from one person to another, meaning that any individual can feel the emotional change of others [34]. When athletic class students constantly stay positive, their positive emotions engage the team. A good interaction within the team creates a support system that makes everyone thrive in the future. Empirical research of Lee, Yang, Lin, and Chen [35] stated that enjoyment and positive emotions are the major sources that encourage students to begin to participate in sports programs. Meeting new people and start a new friendship keep them in the programs. It is evident that engagement in sports provides a great opportunity to develop athletic skills and build positive values [36].

### 5.2. The Relationship between Positive Emotions and Adaptation of School Life

Study findings showed that athletic class students’ positive emotions had positive effects on their adaptation of school life. Findings echoed Fredrickson’s broaden and build theory [11]. The theory describes that positive emotions broadens one’s awareness and encourages exploratory thoughts and actions, and over time this broadened behavioral repertoire builds skills and resources. Resources, like physical and mental health, social resources, positive mind and behaviors, can be particularly useful in dealing with tough times. Results were consistent with previous findings that participation in sports can improve one’s abilities to manage emotions, enhance life experience, and help one find enjoyment in life [37]. Group sports, thus, can provide more opportunities for students to interact with others [36]. More importantly, sports participation and game rules not only optimize student athletes’ chance to develop virtues and characters and to learn social and life skills, but also reduce stress level which certainly can help students’ adaptation of school life [38].

### 5.3. The Relationship between Interpersonal Relationships and Adaptation of School Life

Our study found that high school athletic students’ interpersonal relationships have a positive impact on the adaptation of school life. Hu and Huang [39] stated that athletic class students are full-time students and athletes at the same time, and they are expected to play both roles equally well. However, expectations from school, teachers and parents turn out to be a cause of stress. Results of the present study suggested that interpersonal relationships of high school athletic class students has positive effects on their school adaptation. It revealed that a support system can be established when students have a positive interaction with teachers and parents. Results were consistent with the literature stating that students developing positive interpersonal relationships tend to better adjust to school life [21]. Lo, Guu, and Tseng [13] stated that good interpersonal relationships have influence on individual’s character development and social adjustment, and our study finding is consistent with those studies.

### 5.4. The Mediating Effect of Interpersonal Relationships among Positive Emotions and Adaptation of School Life and Adaptation of School Life

Our study found that high school athletic students’ interpersonal relationships mediated the relationship of positive emotions’ effect on their adaption of school life. As Lo, Guu, and Tseng [13] pointed out, good interpersonal relationships have positive impacts on individual’s character development and social adjustment, therefore, students with positive emotions and good interpersonal relationships can certainly help their adaptation of school life.

## 6. Conclusions, Suggestions and Limitations

### 6.1. Conclusions

The study had explored the effects of high school athletic students’ positive emotions and interpersonal relations on adaption of school life. Study confirmed both positive emotions and interpersonal relations had significant positive impacts on their adaption of school life. In addition, students’ interpersonal relationships mediated the relationship between positive emotions and adaption of school life.

### 6.2. Suggestions

#### 6.2.1. Suggestion for High School Administration

School principals and teachers should encourage students to engage in sports. Gifted students may join the athletic teams while average students may join the after-class programs. Engagement in sports helps students increase positive emotions, build interpersonal relationships and adjust to school environment. Chen and Lin [40] also stated that student athletes need a class environment that encourages equally sports and learning. As these student athletes compete to honor their school, they deserve some academic remedies through a collaborative effort between school, class instructors and athletic coaches. Their academic improvement in turn enhances their interests in learning.

#### 6.2.2. Suggestion for Future Research

The current study was built on the foundation of positive psychology and conducted to verify the relationship among high school athletic class students’ positive emotions, interpersonal relationships, and adaptation of school life. To get a full picture of how positive psychology influences student athletes’ school adjustment, future studies may be conducted to evaluate their life goals, aspiration, and health status.

Moreover, student athlete career development can be a subject for future research. The majority of high school athletic class students have keen interests in becoming professional athletes or athletic coaches; however, they tend to lose the passion for sports once they are admitted to college. A conversation about future career choices can provides a clear path forward for students who might lose sight of life’s possibilities and struggle to make a leap from school to career. Career planning should prepare student athletes for life after high school athletics.

#### 6.2.3. Limitations

This study did not consider different types of sports athletes’ positive emotions and interpersonal relationships’ effect on adaption of school life. Individual and multi players’ sports athletes’ positive emotions and interpersonal relationships’ effects on adaption of school life can be explored separately in the future.

## Figures and Tables

**Table 1:** Reliability analysis and latent variable correlations.

Variable	1.	2.	3.	4.	5.	6.	7.	8.
1. Joy	0.826							
2. Satisfaction	0.622	0.822						
3. Flow	0.610	0.509	0.797					
4. Social skills	0.559	0.562	0.528	0.823				
5. Peer attachment	0.556	0.473	0.600	0.646	0.825			
6. Peer relationship	0.570	0.493	0.627	0.674	0.790	0.830		
7. Learning adjustment	0.502	0.549	0.538	0.604	0.500	0.547	0.797	
8.Teacher-student relationship	0.473	0.545	0.528	0.569	0.577	0.608	0.690	0.867
Composite reliability	0.915	0.862	0.875	0.863	0.894	0.917	0.897	0.938
Cronbach’s α alpha	0.882	0.759	0.808	0.761	0.841	0.887	0.855	0.917

Diagonals represent the average variance extracted (the square root of the average variance extracted in the parentheses) while the other entries represent the correlations.

**Table 2:** Demographic analysis.

Variable	Group	*n*	%	Variable	Group	*n*	%
Gender	male	600	82.8	Grade in school	Freshman	287	39.6
	Female	125	17.2		Sophomore	226	31.2
Year of sport experience	1 year	38	5.2		Senior	212	29.2
	2–3 years.	100	13.8	Sport expertise	Ball sport (basketball, volleyball, baseball)	446	61.5
	4–5 years	167	23.0		Choreography (cheerleading, gymnastics, ballroom dance)	27	3.7
	6 years and more	420	57.9		Equipment-requiring sport (archery, fencing, track and field)	46	6.3
					Competitive sports (taekwondo, judo, martial arts)	206	28.4
